# Prebreeding studies of near-isogenic spring bread wheat lines, differing by presence or absence of the 3R(3D) chromosomal substitution from the triticale cultivar Satu

**DOI:** 10.18699/vjgb-25-85

**Published:** 2025-10

**Authors:** S.N. Sibikeev, I.G. Adonina, A.E. Druzhin, Z.E. Fitileva, O.A. Baranova

**Affiliations:** Federal Center of Agriculture Research of the South-East Region, Saratov, Russia; Institute of Cytology and Genetics of the Siberian Branch of the Russian Academy of Sciences, Novosibirsk, Russia; Federal Center of Agriculture Research of the South-East Region, Saratov, Russia; Federal Center of Agriculture Research of the South-East Region, Saratov, Russia; All-Russian Research Institute of Plant Protection, Pushkin, St. Petersburg, Russia

**Keywords:** triticale Satu, near isogenic lines of bread wheat, 3R(3D) substitution, resistance to leaf and stem rust, influence for productivity and grain quality, тритикале Satu), почти изогенные линии мягкой пшеницы, замещение 3R(3D, устойчивость к листовой и стеблевой ржавчинам, влияние на продуктивность и качество зерна

## Abstract

One of the sources of resistance to leaf and stem rust pathogens for bread wheat is the Australian spring triticale cultivar Satu, which carries highly effective linked SrSatu/LrSatu genes localized on chromosome 3R. However, they are little used in the practical breeding of Triticum aestivum L. The main reason for that is a low level of knowledge regarding the 3R(3D) chromosomal substitution. This paper presents the results of a comparative study of the agronomic value of near-isogenic spring bread wheat siblings, L16 and L17 = Satu/Saratovskaya 70//Saratovskaya 74/3/Saratovskaya 74, differing by presence (L16 (3R(3D))) or absence (L17 (3D3D)) of chromosome 3R from Satu in 2023–2024. The 3R(3D) chromosomal substitution in L16 was detected by cytogenetic analysis combining GISH with labeled Secale cereale genomic DNA and FISH with probes pSc119.2, pAs1. Line L16 is highly resistant to Puccinia triticina and P. graminis, including the Ug99 race. PCR analysis with DNA markers of Sr genes revealed the non-identity of the resistance gene in L16 to Sr genes: Sr2, Sr24, Sr25, Sr28, Sr31, Sr32, Sr36, Sr38, Sr39, Sr47 and Sr57. L16 was inferior to both L17 and the standard cultivar Saratovskaya 76 in terms of 1,000-grain weight. An analysis of productivity elements of the main ear revealed that the 3R(3D) substitution in L16 significantly reduced the length of the ear, increased the density of the ear and did not significantly affect the number of spikelets and the number of grains per ear and the grain weight per ear. The grain protein content in L16 did not significantly differ from its L17 siblings or Saratovskaya 76. Similarly, there were no significant differences in gluten content. However, gluten in L16 was weaker in comparison with line L17 and Saratovskaya 76. According to the complex trait of SDS sedimentation, L16 was inferior to L17, but did not significantly differ from the standard cultivar. According to the alveograph, L16 had significantly lower dough elasticity and flour strength, but in comparison with the standard cultivar, the decrease in flour strength was not significant. L16 showed a higher bread volume than Saratovskaya 76, but did not significantly differ from its L17 sibling. There was no difference in porosity for all three samples. In general, in terms of the complex of agronomically valuable traits, the spring bread wheat line L16 (3R(3D)) requires further work to improve its breeding value.

## Abstract (ru)

Одним из источников устойчивости к возбудителям листовой и стеблевой ржавчин для мягкой пшеницы является австралийский сорт ярового тритикале Satu, несущий высокоэффективные сцепленные гены SrSatu/LrSatu, локализованные в хромосоме 3R. Однако они мало используются в практической селекции Triticum aestivum L. из-за недостаточной изученности влияния этих генов на продуктивность и качество зерна. В настоящей работе представлены результаты сравнительного исследования агрономической ценности почти изогенных линий-сибсов яровой мягкой пшеницы Л16 и Л17, полученных с участием сорта Satu и различающихся наличием (Л16) или отсутствием (Л17 (3D3D)) замещения 3R/3D. Хромосомное замещение 3R(3D) у Л16 выявлено при цитогенетическом анализе, сочетающем GISH c меченой геномной ДНК Secale cereale и FISH с зондами pSc119.2, pAs1. Линия Л16 высокоустойчива к Puccinia triticina и P. graminis, включая линейку расы Ug99. ПЦР-анализом с ДНК-маркерами генов Sr установлена неидентичность гена устойчивости у Л16 генам Sr: Sr2, Sr24, Sr25, Sr28, Sr31, Sr32, Sr36, Sr38, Sr39, Sr47 и Sr57. Урожайность зерна у Л16 в оба года исследований была ниже, чем у Л17 и сорта-стандарта Саратовская 76. По массе 1000 зерен Л16 уступила как Л17, так и Саратовской 76. Анализ элементов продуктивности главного колоса показал, что замещение 3R(3D) у Л16 значимо уменьшило длину колоса, повысив его плотность, и практически не повлияло на количество колосков и зерен в колосе и массу зерна с колоса. По содержанию белка в зерне линия Л16 значимо не отличалась ни от своего сибса Л17, ни от сорта Саратовская 76. Схожим образом не обнаружились значимые различия по содержанию клейковины. Однако клейковина у Л16 была более слабой по сравнению как с Л17, так и с сортом Саратовская 76. По комплексному показателю SDS-седиментации Л16 уступила Л17, но незначимо различалась с сортом-стандартом. По показателям альвеографа у Л16 более низкие упругость теста и сила муки, но по сравнению с сортом-стандартом понижение силы муки незначимое. По объему хлеба Л16 с 3D(3R) имела большее значение, чем Саратовская 76, но незначимо отличалась от своего сибса Л17 с 3D3D. По пористости все три образца не отличались друг от друга. В целом по комплексу хозяйственно ценных признаков линия яровой мягкой пшеницы Л16 (3R(3D)) требует дальнейшей работы по улучшению ее селекционной ценности.

## Introduction

Bread wheat (Triticum aestivum L.) is one of the main food crops. Of the total world grain production, wheat accounts for over 27 %. Bread wheat has the largest areas of crops and is the main food product for a third of the world’s population. A significant erosion of the gene pool of this crop for genes of resistance to disease and pests occurred due to intensive breeding to increase productivity that took place in the 20th century (Dymchenko et al., 1990).

Pathogens have greater genetic variability in their biological characteristics, and two or three epiphytoties and a crop area of 50–100 thousand hectares are sufficient to overcome the resistance gene of the host plant. At present, the main diseases in the bread wheat growing zones continue to be stem, leaf and stripe rust, powdery mildew, leaf and ear septoria, and various types of viral infection. The susceptibility of bread wheat cultivars to diseases leads to huge losses in grain yield and a decrease in bread-making quality traits (Sibikeev, Krupnov, 2007).

Wild relatives of bread wheat have many genes of agronomic interest and can be valuable sources of resistance to diseases, insects, and extreme environmental factors (Sibikeev et al., 2019). To protect bread wheat from pathogens and, first of all, from rust diseases, the resistance genes localized in alien chromosomes and translocations are widely used. Thus, out of 82 identified Lr genes, 39 were transferred from “wild” relatives, and out of 63 Sr genes, 26 were introgressed (McIntosh et al., 2013, 2018, 2022). The following genes were transferred and identified from Secale cereale L.: Sr27, Sr31, Sr50, Sr1RSAmigo (McIntosh et al., 2013), and from triticale: SrSatu, SrBj, SrNin, SrLa1, SrLa2 and SrVen (McIntosh et al., 1995; Adhikari, 1996).

One of the donors of leaf and stem rust pathogens resistance for bread wheat is the Australian spring triticale cultivar Satu, which carries highly effective linked genes SrSatu/LrSatu, localized in chromosome 3R. Moreover, SrSatu is presumably allelic to the Sr27 gene, which is found in many triticale varieties (McIntosh et al., 1995). The SrSatu gene is highly effective against the races of Puccinia graminis f. sp. tritici Erikss. & Henning from the USA, Kenya and South Africa, including the lineage of the Ug99 race, namely TTKSK, TTKST, TTTSK, TRTTF, TTTTF, RKQQC, QTHJC, TPMKS, TKTTF, MCCFC (Rahmatov et al., 2016). In the Russian Federation, the SrSatu gene is effective against the P. graminis populations of the Middle and Lower Volga regions and the Northwestern region (Baranova et al., 2023, 2024). However, SrSatu is extremely rarely used in bread wheat breeding. The main reason for this is insufficient knowledge of the effect the 3R chromosome with the SrSatu/LrSatu genes has on cytological stability, the main traits of grain productivity, and bread-making quality traits.

For use of alien substitutions and translocations in wheat breeding, prebreeding studies are necessary to determine their cytological stability in the genome, the effect on the adaptive properties of plants, as well as on elements of productivity, grain yield and the quality of the final product (Sibikeev, Druzhin, 2015).

The purpose of our research was to determine, based on the results of studying the near-isogenic siblings of spring bread wheat L16 (3R(3D)) and L17 (3D3D), their cytological stability and potential for practical breeding both in terms of effectiveness against rust diseases and in terms of their impact on grain productivity and bread-making quality traits.

## Materials and methods

The material used included near-isogenic siblings of spring bread wheat L16 and L17, which were obtained by crossing the Australian cultivar of spring hexaploid triticale Satu, resistant to leaf and stem rust pathogens, and the cultivars of spring bread wheat Saratovskaya 70 and Saratovskaya 74, susceptible to the indicated types of rust. Pedigree: Satu/Saratovskaya 70 //Saratovskaya 74/3/Saratovskaya 74. Sibling lines L16 and L17 were obtained by the method of forced heterozygotes. They were maintained in the heterozygous state until the seventh generation; then the homozygosity of the sib plants was confirmed over three generations. Both lines belong to the albidum subspecies and differ from each other for their resistance to leaf and stem rust pathogens (L16 is resistant and L17 is susceptible to rust). Thus, the marker trait of the SrSatu/LrSatu genes from triticale in L16 was resistance to leaf and stem rust pathogens. To characterize the grain productivity and the bread-making quality traits, the L16 and L17 lines were compared with each other, as well as with the spring bread wheat cultivar Saratovskaya 76 – this standard was adopted by the State Commission of the Russian Federation for Breeding Achievements Test and Protection in the Saratov region.

Cytogenetic studies. Karyotype analysis of the lines was performed using fluorescent in situ hybridization (FISH) probes based on repeating sequences of pSc119.2 (Bedbrook et al., 1980) and pAs1 (Rayburn, Gill, 1987) on mitotic metaphase chromosomes. Mitotic chromosome preparations were prepared from the root meristem of seedlings according to the method (Badaeva et al., 2017). FISH was performed using the method described in (Salina et al., 2006) with minor modifications. Genomic in situ hybridization (GISH) was performed using labeled S. cereale genomic DNA as a probe in combination with a 10–30-fold excess of unlabeled fragmented T. aestivum DNA according to a previously published work (Schubert et al., 1998). The preparations were analyzed using an Axio Imager M1 microscope (Zeiss, Germany) equipped with a ProgRes MF CCD digital camera (Jenoptik, Germany) using the Isis image analysis program (Meta Systems, Germany). The work was carried out at the Center for Collective Use for Microscopic Analysis of Biological Objects of the Siberian Branch of the Russian Academy of Sciences (Novosibirsk, Russia).

Cytological stability was evaluated by studying the behavior of chromosomes in microsporogenesis in meiosis. Microsporogenesis was studied on temporary squashed preparations. The ears of the L16 and L17 lines were cut before leaving the leaf sheath and fixed in a mixture of 96 % ethanol and glacial acetic acid (3:1). A day after fixation, the material was transferred to 70 % ethyl alcohol, where it was stored until analysis at +2–4 °C. Schiff reagent was used for staining. For each line, 100–200 microsporocytes of meiotic stages (metaphase I and II, anaphase I and II, telophase I and II, tetrads) were analyzed. Slides were analyzed on an Axio Scope A 1 microscope (Carl Zeiss) with N-ACHROPLAN 40×/0.65 and N-ACHROPLAN 100×/1.25 0;1 objectives

Phytopathological studies. Since L17 was selected as a sibling of L16 susceptible to P. triticina and P. graminis, only L16 was used in phytopathological studies. To evaluate the resistance of the L16 line to the stem rust pathogen in laboratory conditions of the All-Russian Institute of Plant Protection, populations collected in 2022 in the Arsk district of the Republic of Tatarstan (from the Nadira cultivar) and in the Samoylovka district of the Saratov region (from the Voevoda cultivar) were used. Virulence analysis of P. graminis f. sp. tritici was carried out using a standard set of 20 differentiator lines (Sr5, Sr21, Sr9e, Sr7b, Sr11, Sr6, Sr8a, Sr9g, Sr36, Sr9b, Sr30, Sr17, Sr9a, Sr9d, Sr10, SrTmp, Sr24, Sr31, Sr38, SrMcN), as well as additional lines with Sr genes (Sr2compl, Sr8b, Sr12, Sr13, Sr15, Sr20, Sr22, Sr25, Sr26, Sr27, Sr28, Sr29, Sr32, Sr33, Sr35, Sr37, Sr39, Sr40, Sr44, SrWLD, Sr24+31, Sr36+31, Sr24+36, Sr7a+12, Sr17+13, Sr7b+18, Sr26+9g and Sr33+5), cultivars Avrora (Sr31) and Khakasskaya (susceptible control). The virulence analysis of pathogen populations from the Nadira and Voevoda cultivars was described by us earlier (Baranova et al., 2023).

The propagation of stem rust pathogen populations and the plants analysis for resistance at the seedling stage were carried out using methods accepted in world practice (Jin et al., 2007). The seedlings reaction to inoculation with a suspension of stem rust pathogen spores was checked on the 12th day using the standard 4-point scale of E.C. Stakman et al. (1962). The resistance/ susceptibility of the sample was determined based on the infection types in two replicates. Plants with infection types “0”, “0;”, “1”, “2” were considered as resistant; plants with infection types “3”, “4”, “Х” were considered as susceptible.

Resistance to race Ug99 (TTKSK) was tested at the adult plant stage in 2023 in the plant pathology nurseries of the International Maize and Wheat Improvement Center (CIMMYT) in Kenya, in the Kenya Agricultural and Livestock Research Organization (KALRO) in Njoro. A modified Cobb scale (Peterson et al., 1948) was used to evaluate plant response. The main distinguishing trait of race Ug99 pathotypes is virulence towards Sr31 genetic carriers. The severity of cultivars with the Sr31 gene being affected in KALRO phytopathological plant nurseries during the vegetation period of 2023 was: for the Prokhorovka cultivar (Sr31) – 60 % (60MSS), for the Yugo-Vostochnaya 2 cultivar (Sr31) – 80 % (80S), for the Saratovskaya 74 cultivar (without identified Sr genes) – 80 % (80S), for the Saratovskaya 70 cultivar (without identified Sr genes) – 40 % (40MSS).

Molecular genetic analysis. For PCR analysis, DNA was isolated from five-day-old wheat seedlings using the cetyltrimethylammonium bromide (CTAB) method (Murray, Thompson, 1980). To identify resistance genes (Sr2, Sr24, Sr25, Sr28, Sr31, Sr32, Sr36, Sr38, Sr39, Sr47 and Sr57), DNA markers recommended for marker-assisted breeding (MAS) were used: Sr2 – CAPS marker csSr2 (Mago et al., 2011); Sr24/Lr24 – STS markers Sr24#12 and Sr24#50 (Mago et al., 2005); Sr25/Lr19 – STS marker Gb (Prins et al., 2001); Sr26 – STS marker Sr26#43 (Mago et al., 2005); Sr28 – DaRT marker wPt-7004-PCR and SSR marker Xwmc332 (Rouse et al., 2012); Sr31/Lr26 – STS marker of SCM9 (Weng et al., 2007); Sr32 – STS marker csSr32#2 (Mago et al., 2013); Sr36 – SSR marker of Xstm773-2 (Tsilo et al., 2008); Sr38/ Lr37 – STS marker of VENTRIUP-LN2 (Helguera et al., 2003); Sr39/Lr35 – STS marker Sr39#22 (Mago et al., 2009); Sr47 – Xgwm501, Xgpw4043 (Faris et al., 2008; Klindworth et al, 2012); Sr57/Lr34 – STS marker of csLV34 (Lagudah et al., 2006).

Amplification was performed on C1000 Thermal Cycler (BioRad) amplifiers, amplification products were separated in 2 % agarose and 8 % polyacrylamide gels stained with ethidium bromide. Isogenic lines and cultivars with known Sr genes were positive controls, the susceptible cultivar Khakasskaya was the negative control, and the PCR mixture without added DNA served as contamination controls. GeneRulerTM 50 bp DNA Ladder (Fementas) was used as a molecular weight marker. Amplification products were visualized using the ChemiDoc XRS+ gel documentation system (Bio-Rad). PCR was performed in two replicates

The evaluation of grain productivity traits, physical and bread making quality traits in the L16, L17 lines and the standard cultivar Saratovskaya 76 was carried out in 2023 and 2024. The experimental material was randomly sown in plots of 7 m2 in three replicates in the experimental field of the Federal Center of Agriculture Research of the South-East Region in Saratov. The seeding rate was 400 grains per 1 m2.

In addition to phenological observations and direct evaluation of grain yield, studies of the elements productivity of the main spike – spike length, number of spikelets and grains, grain weight per spike, spike density, grains for one spikelet (as a general fertility trait) – of the studied lines and the standard cultivar were carried out. These analyses were conducted for 15 spikes of L16, L17 and Saratovskaya 76. Bread-making quality traits were evaluated by the content of crude gluten, the strength of which was determined using the Gluten Deformation Meter IDK-3M (OOO PLAUN), as well as by the Chopin alveograph (Chopin Technologies) traits with the baking of experimental samples of bread. The protein content in grain from the 2023 and 2024 harvests was determined using the Foss Infratec TM 1241 grain analyzer (Foss Analytical A/S).

The meteorological traits of the years of cultivation according to Selyaninov’s hydrothermal coefficient (www. agrometeo.online/articles/gtk.htm, accessed 28.01.2025) by months of the growing season showed the following. In 2023, the hydrothermal coefficient for May was 0.8, in June, 1.1, in July, 0.6 and in August, 0.4. In 2024, the hydrothermal coefficient for April was 0.3, for May, 0.1, for June, 0.8 and for July, 0.1. Of the two years of research, 2023 was the more favorable in terms of meteorological conditions. In both years of research, leaf rust epiphytoties were observed.

The data obtained for the L16 and L17 lines and the standard cultivar Saratovskaya 76 were subjected to a one-way variance analysis with multiple comparisons according to Duncan; an analysis of the genotype–environment interaction was also carried out using the Agros-2.09 breeding and genetic software package (Martynov, 1999).

## Results

**Cytogenetic analysis of spring bread wheat lines L16 and L17**

**Fig. 1.**
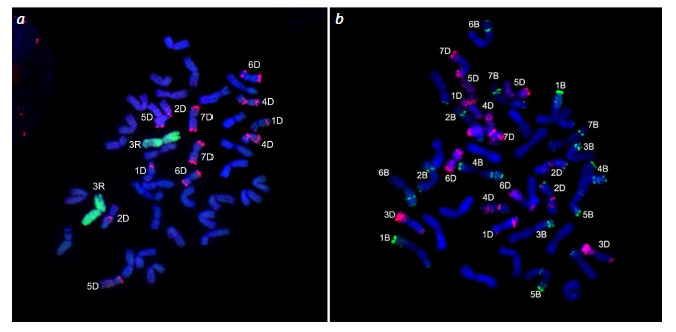
Results of in situ hybridization with different combinations of probes on metaphase chromosomes of spring bread wheat lines L16 (a) and L17 (b). Probes: (a, b) pAs1 (red signal); a – GISH with S. cereale genomic DNA (green signal), b – pSc119.2 (green signal).

Line karyotyping was performed by FISH using a combination of pSc119.2 and pAs1 probes for chromosome identification (Schneider et al., 2003). Cytogenetic analysis of the L16 line revealed the absence of a pair of 3D chromosomes (they are determined by characteristic pAs1 signals) (Fig. 1a) and showed a pair of large chromosomes with bright pSc119.2 signals at the ends of the arms, which is typical for rye 3R chromosomes. Chromosomal substitution 3R(3D) in the L16 line was also confirmed by GISH with S. cereale DNA as a probe (Fig. 1a). Analysis of the L17 line did not reveal any chromosomal rearrangements (Fig. 1b).

**Cytological stability of the L16 and L17 lines**

The main factor limiting the practical application of distant hybrids is their instability, leading to a rapid loss of alien genetic material. This instability is based on disturbances in the meiotic cycle of hybrid plants, causing the formation of non-functional gametes (Orlovskaya et al., 2015). It is known that the genome of distant hybrids and amphidiploids contains systems of genetic meiosis control of different parental species, which not only act independently in the hybrid genome, but also mutually influence each other (Naranjo et al., 1979; Lelley, Larter, 1980; Orlovskaya et al., 2015). In this regard, it is necessary to determine the cytological stability of both distant hybrids and introgressive lines. Since the L16 line is characterized by the 3R(3D) substitution, and chromosome 3D is the carrier of the Ph2 gene (McIntosh et al., 2013), there is reason to expect disturbances in the meiotic cycle. In addition, we conducted a comparative analysis of the meiotic stability in the L16 and L17 lines.

For evaluation of meiosis stability, an integral trait is used – the meiotic index, which is the percentage of normal tetrads to the total number of cells studied. Studies have shown that L16 (3R(3D)) has a meiotic index of 95 %, while L17 (3D3D) has a meiotic index of 94 %, i. e. there are no differences between the lines in this trait. With a meiotic index of 90 % or higher, the plant is cytologically stable, i. e. both lines studied are stable. However, some tetrads of these lines have 1–2 inclusions and triads. In earlier stages of meiosis, asynchrony was found in both lines: earlier divergence of one bivalent in metaphase I, two lagging chromosomes in anaphase I and two chromosomes not included in the nuclei in telophase I. Among the mononuclear pollen, micropollen in the L16 and L17 lines was 1.5 and 2.1 %, respectively.

**Phytopathological analysis of line L16 for resistance to stem and leaf rust pathogens. Identification of stem rust resistance genes using molecular markers**

During the production of near isogenic sibs, the L17 line was selected as stably susceptible to both stem and leaf rust pathogens at all stages of plant growth (infection type IT = 33+) under greenhouse and field conditions. In this regard, the evaluation of resistance to P. graminis f. tritici and P. triticina was carried out on L16 with the substitution of 3R(3D) and the standard cultivar Saratovskaya 76 (Table 1). It should be noted that the analysis of the virulence of the P. graminis f. tritici population from the Favorit cultivar showed that the following genes and their combinations are effective: Sr2compl, Sr13, Sr22, Sr26, Sr27, Sr31, Sr32, Sr33, Sr35, Sr39, Sr24+Sr31, Sr36+Sr31, Sr26+Sr9g, Sr17+Sr13, Sr33+Sr5. The pathogen population from the Favorit cultivar on the line with Sr27 (the gene transferred from S. cereale and localized in the 3R chromosome, widely present in triticale cultivars (McIntosh et al., 1995)) gave reaction type “2” (IT = 2), and from the populations from the Nadira and Voevoda cultivars – “2+” (IT = 2+) and “1” (IT = 1), respectively.

**Table 1.**
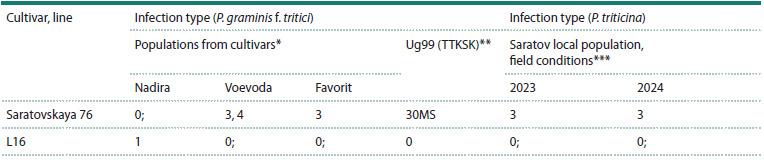
Characteristics of spring bread wheat line L16 and the standard cultivar Saratovskaya 76 for resistance to P. graminis f. tritici and P. triticina in field conditions (natural infection background) and laboratory conditions (artificial infection) * Populations of P. graminis f. tritici collected from spring bread wheat cultivars Nadira (Arsk district of the Republic of Tatarstan), Voevoda (Samoilovka district of the Saratov region) and Favorit (Arkadak district of the Saratov region). Laboratory evaluation at the 3-leaf stage. ** Evaluation of the Ug99 race pathotypes was carried out at Njoro KALRO phythopalogical nursery, Kenya. *** Evaluation was carried out during natural epiphytotics of P. triticina in the experimental field of the Federal Center of Agriculture Research of the South-East Region

As can be seen from Table 1, L16 showed high resistance to all populations of P. graminis f. tritici, including the Ug99 race. Under natural epiphytoties conditions of P. triticina in 2023 and 2024, L16 showed resistance to the local Saratov population. At the same time, the standard cultivar Saratovskaya 76 was susceptible to both populations of P. graminis f. tritici (the exception was the population from the cultivar Nadira IT = 0;) and to P. triticina

High resistance to both rust pathogens, to all populations of pathogens from different parts of Russia and Kenya makes disease resistance in L16 attractive for breeding work. It should be noted that L16 in the Njoro KALRO nursery, Kenya, also showed resistance to the local population of P. striiformis f. sp. tritici West. – 5R, and the Saratovskaya 76 cultivar to – 5M. The results of Sr genes identification in the analyzed L16 line using molecular markers for the Sr2, Sr24, Sr25, Sr28, Sr31, Sr32, Sr36, Sr38, Sr39, Sr47 and Sr57 genes showed their absence.

**Phenology, grain productivity and bread making quality traits in lines L16, L17 and standard cultivar Saratovskaya 76**

**Fig. 2.**
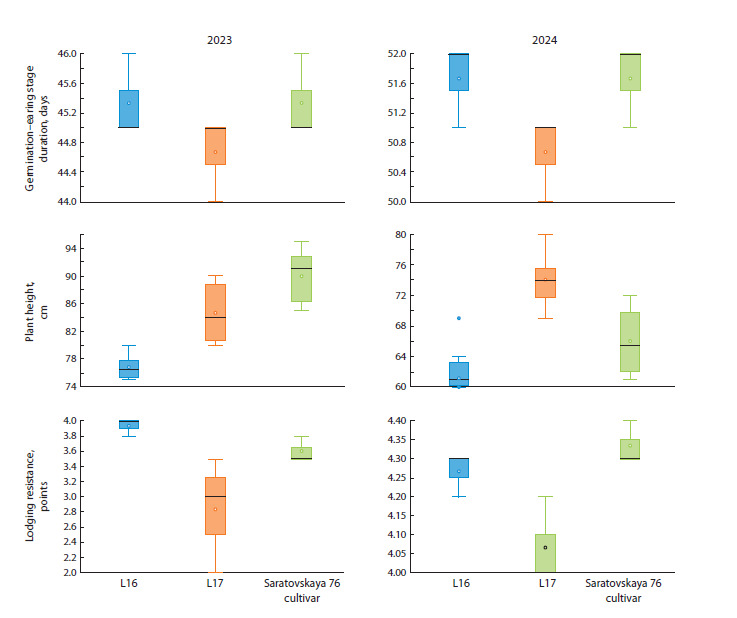
Traits of the germination–earing stage duration, plant height, lodging resistance in the growing seasons of 2023 and 2024 for L16, L17 and the standard cultivar Saratovskaya 76.

Figure 2 shows the traits of the germination-earing stage duration, plant height, lodging resistance in the vegetation seasons of 2023 and 2024 for L16, L17 and the standard cultivar. For the vegetation seasons of 2023 and 2024, the germination–earing stage duration in the sibling lines L16 and L17 and the standard cultivar Saratovskaya 76 was almost the same and the differences were insignificant. Thus, the substitution of the 3D chromosome of bread wheat by the 3R chromosome from the spring triticale cultivar Satu has almost no effect on the duration of the germination–earing period. In 2023, plant height of the L16 line, a sib with the 3R(3D) substitution, was significantly lower than that of its pair with normal 3D3D chromosome composition and the standard cultivar Saratovskaya 76, and in 2024, it was significantly lower than in the L17 line, but was at the level of the standard cultivar.

Thus, the substitution of the 3D chromosome by the 3R chromosome led to a decrease in plant height, which affected the evaluation of lodging resistance in the 2023 growing season, which was significantly higher in the sib with the 3R(3D) substitution compared to the sib pair and the standard cultivar. In 2024, the lodging resistance of L17 was significantly lower than that of Saratovskaya 76, and L16 did not differ from either L17 or the standard cultivar. However, L16 with the 3R(3D) chromosomal substitution exceeded the value of L17 in absolute lodging resistance. Analysis of the genotype–environment interactions between the L16, L17 lines and the Saratovskaya 76 cultivar for the germination–earing stage duration, plant height and lodging resistance showed that these interactions are not significant. Comparisons of L16 and L17 for grain yield and elements of main spike productivity made it possible to identify the effect of alien substitution with the 3R chromosome on these traits (Table 2). It was found out that the 3R(3D) substitution in both years of research reduced grain yield both in comparison with L17 (3D3D) and with the standard cultivar Saratovskaya 76. One of the factors that reduced grain productivity was the lower 1,000-grain weight of L16 in 2023 and 2024 (Table 2).

**Table 2.**
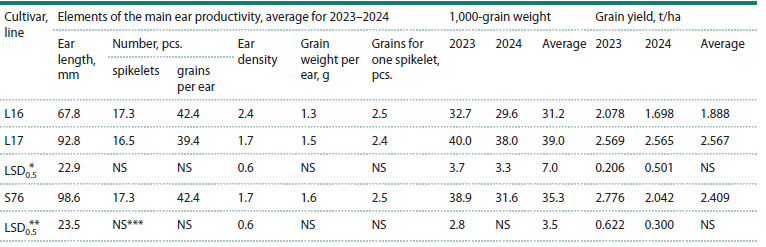
The grain yield, 1,000-grain weight and elements of the main ear productivity of spring bread wheat lines L16, L17 and the Saratovskaya 76 cultivar for the growing seasons of 2023 and 2024 * The least significant difference for the 5 % significance level between L16 and L17. ** The least significant difference for the 5 % significance level between L16 and Saratovskaya 76. *** NS – no difference.

The analysis of the main spike productivity elements revealed that the substitution 3R(3D) reduces the spike length, increasing its density. However, L16 did not differ from L17 and the Saratovskaya 76 cultivar in the number of spikelets, grains, and grain weight per spike. In terms of grain content per one spikelet (a generalized fertility criterion), L16 did not differ from either L17 or the Saratovskaya 76 cultivar. The analysis of the genotype-environment interaction between the L16, L17 lines and the Saratovskaya 76 cultivar for all traits of spike productivity structure, as well as 1,000-grain weight and grain yield, showed that this interaction was not significant.

The analysis of bread-making quality traits showed the following results. Grain protein content in L16 (3R(3D)) did not differ significantly from either its sib L17 (3D3D) or the Saratovskaya 76 cultivar. Similarly, no significant differences were found in gluten content. However, according to the IDK-3M device, L16 gluten was weaker compared to both L17 and Saratovskaya 76 (Table 3).

**Table 3.**
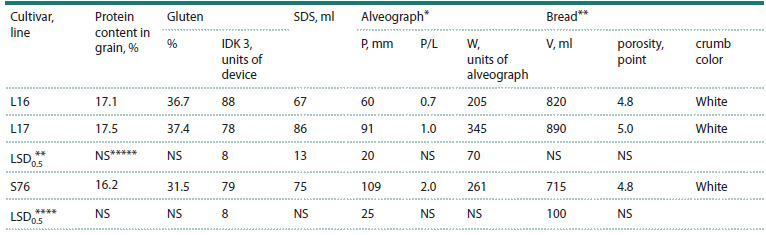
Bread making quality traits in spring bread wheat lines L16, L17 and the standard cultivar Saratovskaya 76 on average for the growing seasons of 2023 and 2024 * Alveograph indices: P – dough elasticity, P/L – ratio of dough elasticity to extensibility, W – flour strength; ** Bread evaluation indices: V – bread volume; *** The least significant difference for 5 % significance level between L16 and L17; **** The least significant difference for 5 % significance level between L16 and Saratovskaya 76; ***** NS – no difference

According to the SDS sedimentation indices, which characterize physical dough properties, L16 was inferior to L17, but did not differ significantly from the standard cultivar Saratovskaya 76. For the alveograph indices, L16 had significantly lower dough elasticity and flour strength, but compared to the standard cultivar, the decrease in flour strength was insignificant (Table 3). L16 had a higher bread volume than Saratovskaya 76, but did not differ significantly from its sibling L17. In terms of porosity, all three samples did not differ significantly from each other, but the highest score (5.0) was found in the L17 line. The substitution 3R(3D) did not change the color of the bread crumb, L16 (3R(3D)) had a white crumb, like L17 (3D3D) and Saratovskaya 76.

## Discussion

As noted above, triticale cultivars are attractive among breeders because of a set of valuable agronomic traits for breeding bread wheat, including disease resistance genes. Thus, the following genes of resistance to the stem rust pathogen have been identified in triticale: Sr27, SrSatu, SrBj, SrNin, SrLa1, SrLa2 and SrVen (McIntosh et al., 1995; Adhikari, McIntosh, 1998). Moreover, the SrBj and SrVen genes control moderate susceptibility at the seedling stage in field conditions, and the SrLa1 and SrLa2 genes determine resistance at the seedling stage both in field and in greenhouse conditions (Adhikari, McIntosh, 1998). The Sr27 and SrSatu genes control resistance throughout the growing season of plants (Singh, McIntosh, 1988). Despite the Sr27 and SrSatu genes being considered as allelic (Singh, McIntosh, 1988), they differ in their effectiveness against the stem rust pathogen. Currently, in the Saratov population, there is an increase in the content of virulent pathotypes of P. graminis f. tritici to the Sr27 gene (in 2016 – 10 %, in 2019 – 20 %, in 2020 – 90 %), while pathotypes virulent to SrSatu are not detected (Kon’kova, 2021)

The results of our studies of the SrSatu gene in L16 confirm the conclusions about its effectiveness. Moreover, its efficiency against the population of P. graminis f. tritici from the Nadira cultivar collected in Tatarstan was revealed, IT = 1, while in the line with Sr27, IT = 2+. These results coincide with the data of testing other lines of spring bread wheat L968 = Satu/S70// S74/3/S70/4/S70 and L935 = Satu/S70//S70 with 3R(3D) from crossings of the triticale cultivar Satu with spring bread wheat cultivars bred by the Federal Center of Agriculture Research of the South-East Region. These lines showed a reaction type of “0” to the population from the Nadira cultivar (Baranova, unpublished data) and a rating of “–0” to the Ug99 race of P. graminis f. tritici (Baranova et al., 2024). In our studies, the L16 line was also resistant to the Saratov populations of P. triticina both during the selection of sibs with 3R(3D) and 3D3D chromosomal composition and in the field experiments in 2023 and 2024.

Thus, the SrSatu/LrSatu genes in chromosome 3R of the L16 line are highly effective against populations from the Lower and Middle Volga regions of P. graminis f. tritici and P. triticina, as well as against the Ug99 pathotypes of P. graminis f. tritici. Consequently, when chromosome 3R was transferred from the triticale cultivar Satu to the genotypes of spring bread wheat line L16, the expression of the SrSatu/ LrSatu resistance genes was not disrupted. Our attempts to identify Sr genes in the analyzed L16 line using molecular markers for the Sr2, Sr24, Sr25, Sr28, Sr31, Sr32, Sr36, Sr38, Sr39, Sr47 and Sr57 genes showed their absence. Thus, it was shown that L16 carries its own (SrSatu) unidentified resistance gene. Unfortunately, to date, there is no DNA marker for the Sr27 and SrSatu genes (МcIntosh et al., 2013).

In our studies of cytological stability in lines L16 and L17, a number of disturbances during the passage of meiosis phases were revealed. However, according to meiotic indices in L16 (3R(3D)) and L17 (3D3D), 95 and 94 %, respectively, these lines are characterized as stable. There are grounds to assume that the absence of the Ph2 gene in L16 (nullisomal state of the 3D chromosome) was compensated by the presence of the 3R chromosome of S. cereale, and in terms of meiosis stability, L16 did not differ from L17.

Unfortunately, in the literature available to us, we did not find a data about the effect of the 3R chromosome from the triticale cultivar Satu in the genotypes of spring bread wheat on grain productivity and the bread-making quality traits. However, the effect of the 3R chromosome from the line 86-741 (F6 hexaploid triticale Guangmai 74 (AABBRR)/Fan 6 (bread wheat)) was studied. The authors studied 185 F8 recombinant inbred lines from crossing bread wheat cultivar Chuanmai 42 with line 86-741. Chromosome 3R was identified by FISH and GISH methods (Wan et al., 2023). The authors found out that the 3R(3D) substitution significantly reduces grain yield, 1,000-grain weight, number of ears per plant, grain weight per ear and has a neutral effect on the number of grains per ear (Wan et al., 2023).

Our studies also noted a decrease in 1,000-grain weight and grain yield, but a neutral effect on grain weight per ear, number of grains per ear, grain content per one spikelet, and a significant increase in ear density. Thus, there is some discrepancy in the effect of 3R(3D) on grain weight per ear. This may be due to differences in the genotype of bread wheat in which the 3R(3D) substitution was studied. In addition, it is necessary to take into account the effect of nullisomy for 3D. Normally, 3D is a carrier of the dominant spherococcoid gene S-D1a (McIntosh et al., 2013), respectively, the null state of this chromosome determines the recessive state of this gene – S-D1b. It is known that the S-D1b gene has a pleiotropic effect, which reduces plant height, ear length, 1,000-grain weight, and increases ear density (Sears, 1947; according to: Salina et al., 2000). All these traits were detected in the L16 line (3R(3D)). Based on this, there is reason to expect that the morpho-biological traits in L16 are formed under the combined influence of the recessive state of the S-D1b gene and the direct action of the 3R chromosome.

The absence of 3D and the presence of the 3R chromosome in L16 affected the bread-making quality traits. Basically, these traits worsened in comparison with sibling line L17. The evaluations of SDS sedimentation, dough elasticity and flour strength decreased significantly. An insignificant decrease was noted for other traits: protein content in grain, gluten content and strength (according to the IDK-3M device), elasticity to dough length ratio (P/L), bread volume and porosity.

## Conclusion

The spring bread wheat line L16 carries highly effective genes for resistance to leaf and stem rust pathogens, which are attractive for breeding for immunity to common wheat pathogens in the Russian Federation. However, in general, the spring bread wheat line L16 (3R(3D)) requires further work to improve its set of economically valuable traits. This is possible by reducing the amount of alien gene material, i. e. by obtaining recombinations or translocations between bread wheat chromosomes and the 3R chromosome, as well as by selecting a bread wheat genotype that will compensate for the negative impact of the rye chromosome on grain productivity and the bread-making quality traits.

## Conflict of interest

The authors declare no conflict of interest.
